# Determining the Failure Rate of Direct Restorations—Chart Review versus Electronic Health Record Reports

**DOI:** 10.3390/dj12080250

**Published:** 2024-08-08

**Authors:** Priyal Patel, Utsavi Kapadia, Janhvi Vyas, Sahil Mhay, Romesh P. Nalliah

**Affiliations:** 1 Office of Patient Services, University of Michigan School of Dentistry, Ann Arbor, MI 48109, USA; ukapadia@umich.edu (U.K.); sahil_mhay2000@yahoo.com (S.M.); 2 Faculty in Periodontics and Oral Medicine Department, University of Michigan School of Dentistry, Ann Arbor, MI 48109, USA; jrvyas@umich.edu; 3 University of Michigan School of Dentistry, Ann Arbor, MI 48109, USA

**Keywords:** restoration failure, dental school setting, failure rate of amalgam–composite restorations, clinical performance of amalgam and composite restorations, reasons for dental restoration failure, failure rate of restoration, academia, anterior versus posterior composite restoration, reason for failure, dental documentation, university settings, importance of documentation

## Abstract

Amalgam and composite restorations are used to treat minor dental issues. University of Michigan, School of Dentistry Electronic Health Record (EHR) reports show a 2.31% failure rate for amalgam and 1.14% for composite. Our study aims to determine the true failure rates through manual EHR chart reviews. Patient data from the University of Michigan School of Dentistry were utilized—216 amalgam restorations from 2020 to 2022 and 350 composite restorations in 2021 were searched. We defined *failure and retreatment* as replacing a restoration with the same material and *failure and alternate treatment* as replacing restoration with an alternative treatment within one year. The *failure rate* refers to a combination of replacement with the same and alternative treatment material within one year. For Amalgam: 1.85% failed and were retreated; 7.87% failed and were received an alternate treatment. Composite: 9.71% failed and retreated; 2.86% failed and received alternate treatment. In total anterior composite: 10.5% retreated, 2.6% failed; posterior composite: 9.1% retreated, 3.0% failed. Our study revealed higher restoration failure rates than the reports extracted in the EHR. This highlights the need to foster a culture of precise documentation to align EHR reports with hand-search findings.

## 1. Introduction

Dental amalgam has been the preferred material for treating caries in posterior teeth [1,2] for centuries. According to the US Food and Drug Administration, amalgam has been deemed safe and effective for dental restoration [3]. In a study by Jardim et al., 172 restorations were evaluated to investigate if material—amalgam and composite—impacted the restoration’s survival over five years. The 5-year survival analysis revealed that amalgam and composite restoration had a similar success rate regardless of the technique used to remove the caries [4].

However, in recent years, a discernible shift has occurred in dentistry toward a preference for dental restorations that prioritize aesthetics [1]. Buonocore’s advent of adhesive dental materials in 1955 led to a giant leap in restorative dentistry [5]. Thus, a minimally invasive philosophy was adopted in dentistry in the early 1970s [5,6]. While modern restorative materials, notably resin composites, have emerged as viable substitutes for dental amalgam, they face challenges in matching several of the advantageous attributes associated with amalgam, including its ease of manipulation and durability [7]. The shift in the oral health paradigm toward preventive intervention rather than operative intervention may have also contributed to the popularity of composite materials that can be bonded and may permit more conservative tooth preparation [8].

In 2013, the Minamata Convention on Mercury proposed a phase-down of mercury-containing products like dental amalgam [9] to address mercury pollution [10,11]. Ratified in 2017, the convention supported research into alternative restorative materials. Alongside mercury pollution concerns, there has been an ongoing trend for resin composite restorations over amalgam for extensive cavity preparations [9,10,11,12,13].

Over the past decade, dental composite materials have significantly improved clinical performance due to polymerization techniques and the advancements of binder incorporation. These enhancements provide better strength, wear resistance, and translucency, making composites a widely utilized restorative material in modern dentistry [14]. However, amalgam and resin composite restoration failure are standard, and their replacement consumes over half of general dental practice services [15]. Several factors contribute to restoration failure, including the type of material used, the dentist’s experience, and the position and type of tooth [16,17]. Clinical studies indicate higher failure rates and decreased durability in dental adhesive restorations than dental amalgams, primarily due to secondary caries and restoration fractures. Factors such as reduced antimicrobial activity, polymerization shrinkage, plaque accumulation, microleakage, and gap formation increase the risk of secondary caries [18].

According to the study by Opdam et al., posterior resin composites completed by students were evaluated for survival over five years. A total of 382 patients were seen, with 703 posterior resin composites completed; 94 restorations had failed due to caries, fracture, lack of proximal contact, and endodontic treatment. The annual failure rate was deemed to be 2.8%, with the survival rate at 87% [19]. A prospective cohort study was conducted with 226 dental practitioners who completed 6218 direct amalgam and composite restorations in 3855 patients. Among all the restorations, the failure rate was 6.2% (386) for the restorations. It was noted that tooth type and material were not associated with the longevity of the restoration. However, the patient’s age was significantly associated with the failure rate (*p* < 0.0001). Individuals 65 and older had a 12% failure rate, compared to 5% in children [20]. Moreover, another study compares the durability of amalgam and composite restoration within posterior teeth in a randomized controlled trial. Among the 472 patients, half were randomly assigned to receive resin-based composite restorations and the other half to receive amalgam restorations. Like our study, failure is defined as “restorations needing replacements”. At baseline, patients received a total of 1748 restorations, and 10.1% of the restorations failed. The main reason for the failure of both materials was secondary caries. It was even noted that within the composite group, the risk of secondary caries was 3.5 times higher than in amalgam restorations [16].

Data on the longevity of composite restorations in posterior teeth have been widely explored. The primary causes of restoration failure in posterior teeth are caries development and fracture, with secondary caries being the main reason for composite restoration failures in high caries risk patients [16,19]. The survival rate in anterior teeth has been even higher than in posterior teeth. However, aesthetic reasons are the leading cause of composite restoration failures [21,22]. An 8-year retrospective study conducted in Brazil found that most data on the longevity of composite restorations available in the literature are from studies in which experienced and trained dentists placed the restorations in low-risk patients with a high socioeconomic level [21].

Our research is focused on determining an accurate failure rate of amalgam and composite restorations at the University of Michigan, School of Dentistry. We conducted a comprehensive manual search of dental records of patients receiving amalgam or composite restorations to achieve this goal. This approach involved meticulously examining patient records and treatment histories to identify instances where amalgam and composite restorations have yet to perform as intended or have required further attention. This detailed data analysis aims to provide an accurate and comprehensive understanding of the success or failure of amalgam and composite restorations in dental educational settings.

## 2. Materials and Methods

Our study was, primarily, a quality assurance project. We first extracted chart numbers for all direct restorations. Then, we manually searched the charts for information related to success and failure of every amalgam restoration delivered. For composite restorations, we identified cases that were placed between 5 January 2021 and 30 March 2021 and monitored for failure for 12 months. Hence, the study period was from 5 January 2021 to 30 March 2022. Similarly, for amalgam, we identified cases that were placed between 8 September 2020 and 27 July 2022. Hence, the study period was from 8 September 2020 to 27 July 2023. All of these restorations were completed at the University of Michigan, School of Dentistry by students in the predoctoral dental clinics under the supervision of licensed faculty clinicians.

With three calibrated reviewers, a manual record search of 216 amalgam restorations and 350 consecutive composite restorations was reviewed during the respective study periods in this retrospective study. The 2-year and 3-month periods represented the date of initial placement of amalgam and composite restorations, respectively. As this was a quality assurance study, we reviewed all composite cases placed over 3 months and reached 350 cases. But it took 2 years to reach 216 cases for amalgams because they are delivered less frequently. The manual review process involved reviewing the notes to determine if patients had expressed satisfaction/dissatisfaction with their restoration, if restorations had been retreated with the same material or treated with an alternative material within one year, and if they had been appropriately documented. The procedures not properly documented would not have been identified in the initial EHR report. When there was disagreement between reviewers, they met with a fourth reviewer. They discussed the case to determine how to classify it.

The primary focus of this evaluation was to identify instances of retreatment and failure. In this quality assurance study, “failure and retreatment” was defined as where a direct restoration failed and the tooth was treated with the same material within one year. Also, “failure and alternate treatment” was characterized by the replacement of the restoration in a different way—either with a different direct restoration material, or an indirect restoration or extracted and replaced in another way within the same one-year period. Therefore, the term “failure rate” refers to a combination of replacement with the same material and an alternative treatment within one year. University of Michigan’s Committee on Human Subjects Research reviewed our study protocol and determined our study as “not regulated”(HUM00253928).

## 3. Results

### 3.1. Amalgam Restorations

**Figure 1:**
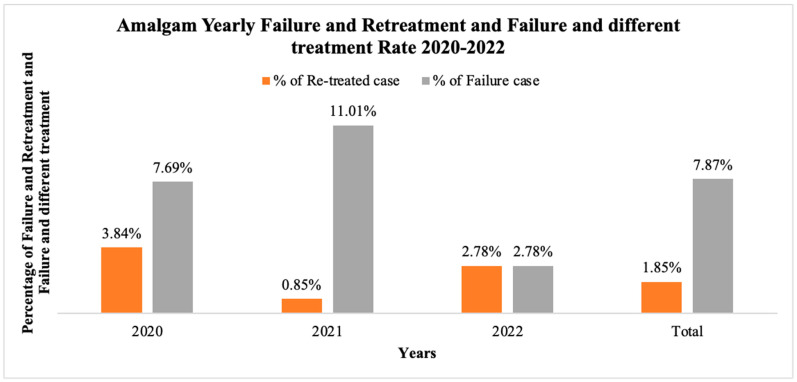
Amalgam yearly failure and retreatment, and failure and different treatment rate 2020–2022.

Only a few amalgam restorations are placed every month at the University of Michigan, School of Dentistry and, therefore, we extended the study period for amalgam restorations. Over the span of two years, from 8 September 2020 to 27 July 2022, a total of 216 amalgam restorations were completed. Within one year of placement, 4 (1.85%) cases had failed amalgam restoration and the tooth was treated with the same material again, and 17 (7.87%) cases replaced the restoration in a different way—either with a different direct restoration material, or an indirect restoration or extracted and replaced in another way (Figure 1).

### 3.2. Composite Restorations

**Figure 2:**
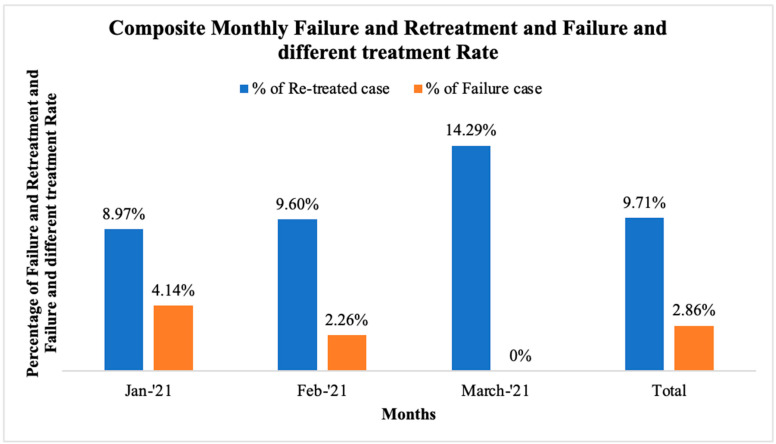
Composite monthly failure and retreatment, and failure and different treatment rate January 2021–March 2021.

Between 5 January 2021 and 30 March 2021, there were 350 composite restorations completed. Within the study period of one year, 34 (9.71%) cases had composite restoration failed and the tooth was treated with the same material, and 10 (2.86%) cases replaced the restoration in a different way—either with a different direct restoration material, or an indirect restoration or extracted and replaced in another way (Figure 2). There were 152 (43.43%) anterior and 198 (56.57%) posterior restoration cases among the 350 composite patients. Sixteen (10.52%) cases had to be retreated with a composite, and four (2.63%) cases were replaced with an alternative treatment among anterior restorations. Meanwhile, among posterior restorations, 18 (9.09%) cases had to be retreated by composite, and 6 (3.03%) were replaced with an alternative treatment (Table 1).

Our chart reviews indicated that secondary caries—which accounts for 16 cases (36.4%) of failures in composite restorations—was the most common cause of failures in composite restorations. Fracture is the second most frequent cause of failure, which accounted for 11 cases (25%) among composite restorations. In addition, 17 cases (38.6%) in composite restorations due to open margin, sensitivity, debonded restoration, pulpal disease, shade change, and other treatment plan factors (Table 2).

Additionally, our data show that the primary causes of failure within amalgam restorations were seven cases (33.3%) of secondary caries and seven cases (33.3%) of restoration fractures. In addition to that, factors such as open margins, sensitivity, lack of retention form, pulpal disease, and other treatment plan issues contributed to seven cases (33.3%) of restoration failures in amalgam restorations (Table 2).

## 4. Discussion

Both amalgam and composite restorations had higher failure rates than reported in EHR data (2.31% for amalgam and 1.14% for composite). Our study’s total failure rate (including retreatment and failures) was 9.72% for amalgam and 12.57% for composite restorations. These findings emphasize the critical need for precise documentation and reporting practices in dental school clinics. If EHR reports consistently underestimate the true failure rates, it may lead to a false sense of security regarding the performance of restorative procedures, potentially impacting patient care and curricular gaps. Dental schools should prioritize training students and faculty in accurate dental coding and documentation practices to ensure that EHR reports align with clinical outcomes [23]. The higher-than-anticipated failure rates in amalgam and composite restorations highlight the need for vigilance and continuous quality improvement in dental education and practice. Identifying contributing factors related to technique, materials, or patient characteristics is essential for informing curriculum adjustments and faculty training to better prepare students for real-world dental challenges [23].

Our study also found a high failure and retreatment rate for composite restorations, which is 9.71%. During this study, the dental school clinics had 4 or fewer dental assistants supporting 118 predoctoral dental chairs—the opportunity for four-handed dentistry with an experienced assistant was limited and this may have contributed to inadequate moisture control and restoration failure. Junior students often volunteer to assist; however, with their lack of experience, they may actually have created more risk of failure by distracting the student provider (who also had to instruct the junior student) and introducing errors at the dental assistant level (such as errors in moisture control).

Our study has major implications for predoctoral dental clinics, including curriculum and calibration efforts. For instance, the implementation of mandatory use of a rubber dam for all students in clinics may significantly address moisture contamination concerns [24]. Rubber dam utilization will assist novice providers in maintaining a dry field and may enhance the longevity of restorations. To our knowledge, this is the first manual chart review of quality performed on completed composite and amalgam restorations in a predoctoral clinical setting. Evidence has shown that, since students are not experienced, the longevity of composite resin restorations may be reduced in dental school clinics [19]. According to Opdam, N. J. M et al.’s study reported that dental students could restore composite restorations in posterior teeth with an acceptable mean annual failure rate [19]. However, the scarce studies with inexperienced operators showed higher annual failure rates (1.7% to 2.8%) [19,25] compared to experienced dentists (1.0% to 1.5%) [26]. Our study found that the failure rate (which we defined as the total of failure and retreatment plus failure and alternate treatment) was 9.72% for amalgam and 12.57% for composite restorations. According to Baldissera RA et al., anterior composite restorations performed slightly better than posterior restorations [22]. A prior study indicated that, after a 3-year interval, there was a higher rate of satisfactory restorations in the anterior region compared to the posterior region when conducted by undergraduate students [22]. However, our findings contradict previous research. Table 1 demonstrates that the total failure rate of anterior restoration (13.15%) is higher than that of posterior composite (12.12%) restoration.

According to Baldissera RA et al., the main reasons for failure are aesthetics for anterior and fracture for posterior composite restorations [22]. Secondary caries and marginal discoloration were the most prevalent reasons for posterior resin composite restoration replacement [1]. Similarly, our study found that secondary caries is the main reason for restoration failure. At the same time, fracture was the second most common reason for failure. The higher loading stresses on posterior teeth may contribute to an increased risk of fatigue in the restorative material [18]. Baldissera RA et al. reported that restorations involving a higher number of surfaces are more likely to fail, mainly due to fractures such as posterior restorations failing in the long term due to fracture and marginal breakdown, and anterior restorations failed more due to aesthetics and anatomic form with fracture also present [22]. Previous research has shown that class III restorations, often surrounded by enamel and positioned in a low-stress-bearing location, last longer than class IV restorations [22]. A retrospective study on posterior composite restorations found secondary caries was the primary cause of failure in high caries risk patients, while fracture was predominant in “occlusal-stress-risk” patients [22]—and these risk factors can be predicted. Table 2 shows that the main reasons for composite restoration failure in our study are secondary caries (36.4%), fracture (25%), and other factors (38.6%) such as open margin, sensitivity, debonded restoration, pulpal disease, shade change, and other treatment plans, while for amalgam restoration, the causes of failure are secondary caries (33.3%), fracture (33.3%), and other factors (33.3%) like open margin, sensitivity, absence of retention form, pulpal disease, and other treatment plans.

Our study found that the actual failure rate is higher when compared with EHR reports. In the digital age, electronic health records (EHRs) have emerged as a game-changing technology in healthcare. Dental clinics are no exception, as they, too, have embraced the transition from traditional paper-based records to electronic systems. Although EHRs have many benefits, they can only be utilized with precise record-keeping and reporting [23,27,28]. Dental records facilitate communication among healthcare providers, support care quality evaluations, and enhance patient care safety and effectiveness [27]. In our study, the data analysis revealed the underreporting of amalgam and composite replacements in EHR reports, thus creating a misleading impression of the effectiveness of restorative procedures and materials. Regularly auditing dental records and education to providers about the risks of miscoding may help ensure the administration has accurate information on patient outcomes when making decisions. This quality assurance process guides enhancing practice operations and encourages internal discussions to prevent future errors [29]. According to Charangowda BK et al., the production, retention, and release of clear and precise patient records constitute a fundamental aspect of the dentist’s professional responsibilities. A practicing dentist should have a good understanding of dental records, as they have legal implications for insurance and consumerism in addition to forensic applications [28].

EHRs can facilitate the electronic transmission of health information, resulting in better, safer, and more effective patient care. However, using the wrong code or underreporting can lead to serious consequences. EHR systems can be incredibly powerful resources for providers but are not risk-free. Proper measures must be implemented to accurately understand the quality assurance measures for predoctoral students in an academic setting.

## 5. Conclusions

Our study provides beneficial insights into the true failure rates of dental amalgam and composite restorations within a dental school clinic setting. The higher failure rates in restorations compared to the reports extracted from the EHR have implications for curriculum and faculty calibration and foster a culture of precise documentation to align EHR reports with hand-search findings. By addressing this issue, dental professionals can improve patient care quality and dental education.

## Figures and Tables

**Table 1:** Composite retreatment and failure cases based on anterior and posterior teeth.

Composite	Anterior	Posterior
Total patients	152 (43.43%)	198 (56.57%)
% of failure and retreatment cases	16 (10.52%)	18 (9.09%)
% of failure and different treatment cases	4 (2.63%)	6 (3.03%)
% of total failure rate	20 (13.15%)	24 (12.12%)

**Table 2:** Reasons for amalgam and composite restoration for failure and retreatment, and failure and different treatment cases.

Reason for Failure and Retreatment, and Failure and Different Treatment	Amalgam	Composite
Secondaries caries	7 (33.3%)	16 (36.4%)
Fracture	7 (33.3%)	11 (25%)
Other (open margin, sensitivity, absence of retention form, pulpal disease, debonded restoration, shade change, other treatment plans)	7 (33.3%)	17 (38.6%)

## Data Availability

The data presented in this study are available on request from the corresponding author due to privacy and ethical restrictions.
